# Twice the Leak: Managing CSF Fistulas in a Recurrent Thoracic Arachnoid Cyst—A Case Report

**DOI:** 10.3390/reports8030152

**Published:** 2025-08-21

**Authors:** Federica Bellino, Leonardo Bradaschia, Marco Ajello, Diego Garbossa

**Affiliations:** Neurosurgery Unit, Department of Neuroscience “Rita Levi Montalcini”, A.O.U. Città della Salute e della Scienza, University of Turin, 10127 Turin, Italy; federica.bellino@unito.it (F.B.);

**Keywords:** pseudomeningocele, fenestration, type I, clipping, percutaneous repair, epidural

## Abstract

**Background and Clinical Significance**: Spinal arachnoid cysts are rare lesions that may become symptomatic through progressive spinal cord compression. We present a complex case of a thoracic extradural SAC in a 17-year-old male, managed through a stepwise, multidisciplinary approach. **Case Presentation**: The patient presented with progressive lower limb weakness, right knee paresthesia, and urinary hesitancy following physical exertion. MRI revealed a large posterior extradural SAC extending from T2–T3 to T8, with associated spinal cord compression. Initial management involved T8 laminectomy and cyst fenestration under intraoperative neurophysiological monitoring, with partial clinical improvement. However, early recurrence with pseudomeningocele formation prompted a second surgery, including external CSF drainage. Persistent cerebrospinal fluid (CSF) leakage led to targeted epidural blood patching, followed by temporary stabilization. Due to continued cyst enlargement and spinal cord compression, definitive surgical repair was undertaken: fistula clipping at T3 and embolization with platinum coils inside the cystic cavity, combined with a new blood patch. This novel technique resulted in radiological improvement and clinical stabilization. **Conclusions**: This case highlights the diagnostic and therapeutic challenges of managing symptomatic extradural SACs, particularly in young patients. Our experience underscores the utility of a staged approach involving surgical decompression, neuroimaging-guided interventions, and definitive dural repair. The combination of fistula clipping and coil embolization may offer a promising strategy for refractory cases, potentially reducing recurrence and preserving neurological function.

## 1. Introduction and Clinical Significance

Spinal arachnoid cysts (SACs) are rare but clinically relevant lesions, accounting for approximately 1–3% of all intraspinal masses. These cerebrospinal fluid (CSF)-filled sacs arise from the arachnoid membrane and may be located intradurally, extradurally, or in both compartments. Although often asymptomatic, SACs can cause a range of neurological symptoms when they compress the spinal cord or nerve roots. The thoracic spine is the most commonly affected region, with cysts typically positioned dorsally or posterolaterally to the spinal cord.

While asymptomatic cysts may be managed conservatively, surgical intervention is generally indicated for symptomatic cases. Complete resection is preferred due to its lower recurrence rate, although cyst fenestration or CSF shunting may be considered when excision is not feasible.

Herein, we present a rare case of a recurrent, symptomatic thoracic SAC with both intradural and extradural components, complicated by dual CSF fistulas in an adolescent patient. We discuss the clinical presentation, radiological features, surgical approach, and postoperative course.

## 2. Case Presentation

### 2.1. First Admission

A previously healthy 17-year-old male began experiencing right knee paresthesia and gradually worsening right foot weakness in September 2023, shortly after engaging in intense physical exercise during gym activities. Over the subsequent months, he developed gait instability and urinary hesitancy, without frank incontinence. Due to worsening symptoms, he presented to the emergency department of our institution in February 2024.

Neurological examination revealed marked right foot dorsiflexion weakness (Medical Research Council [MRC] grade 1/5), moderate plantarflexion weakness (MRC 3/5), and hyperactive bilateral patellar reflexes with right-sided reflexogenic spread. Bilateral Achilles clonus and a right Babinski sign were also noted. Sensory examination, including perineal sensation, was preserved.

**Figure 1:**
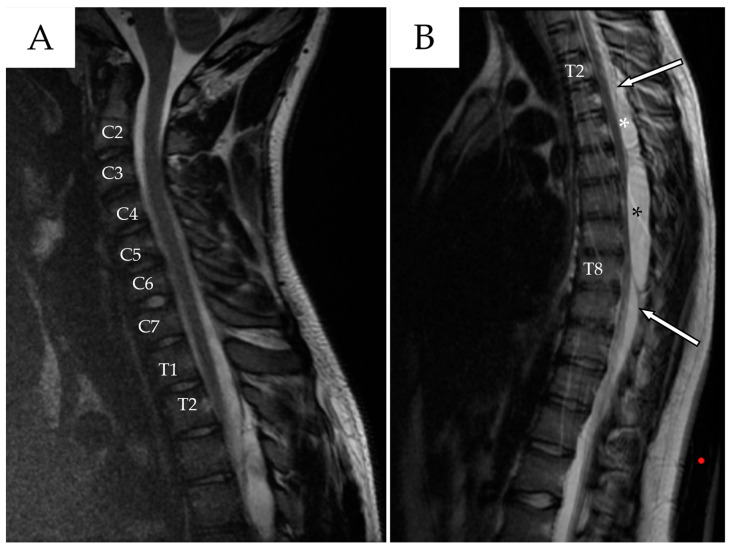
(**A**) Cervical spine ceMRI, sagittal section, TSE T2-WI. The cranial portion of the SAC is visible at the T2 level. To facilitate counting, the cervical vertebrae and the first two thoracic vertebrae are marked with their corresponding names. (**B**) Thoracic spine ceMRI, sagittal section, TSE T2-WI. The SAC is visible in its entirety, composed of a smaller cranial portion (T2–T4, white asterisk) and a larger caudal portion (T5–T8, black asterisk). The dural sac is easily identifiable (white arrows with black outlines), confirming the extradural location.

**Figure 2:**
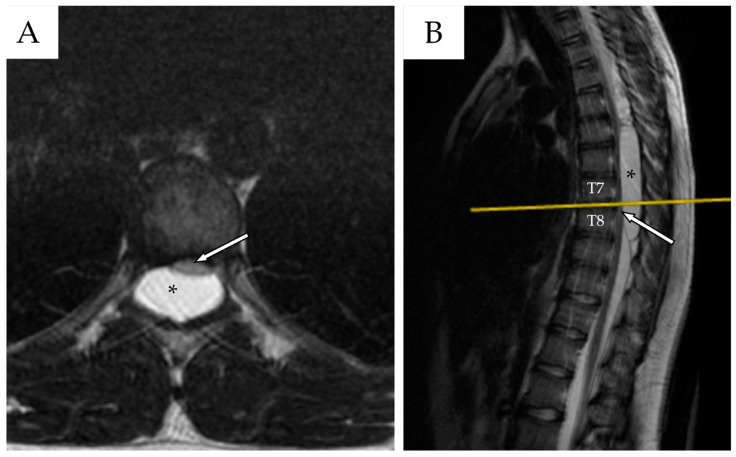
(**A**) Thoracic spine ceMRI, axial section, TSE T2-WI. The section is taken at the highest point of spinal cord compression (T7–T8), with early signs of radiological myelopathy (white arrow with black outlines). The spinal cord is dislocated anteriorly against the vertebral posterior wall. The SAC is marked with a black asterisk. (**B**) The yellow line indicates the level at which the axial section in (**A**) was obtained.

Whole-spine contrast-enhanced Magnetic Resonance Imaging (ceMRI) demonstrated a posterior, extradural intraspinal lesion extending from T2 to T8, causing significant compression of the dural sac and spinal cord (Figure 1). The lesion was formed by two components: a cranial one (T2–T4) and a larger caudal one (T5–T8). The area of maximal spinal cord compression was identified at T7–T8, where subtle intramedullary T2 hyperintensity was observed, suggestive of early spinal cord changes consistent with possible myelopathy (Figure 2). The lesion appeared hyperintense on T2-weighted images, isointense with CSF, and showed no contrast enhancement—findings consistent with a spinal arachnoid cyst.

No features typically associated with arachnoiditis—such as nerve root clumping, leptomeningeal enhancement, or intrathecal adhesions—were present on MRI. Moreover, there was no prior history of trauma, infection, or spinal instrumentation, further supporting a congenital origin of the cyst.

Given the longitudinal extent of the arachnoid cyst along the spinal canal, complete resection would have required a multilevel laminectomy from T2 to T8. This approach carried the potential need for subsequent spinal stabilization and would have necessitated a large surgical exposure. Considering the patient’s young age (17 years), we opted instead for cyst fenestration as a more conservative yet effective strategy.

The patient therefore underwent emergent thoracic decompression with a T8 laminectomy and cyst fenestration, performed under continuous intraoperative neurophysiological monitoring (IONM). Upon completion of the laminectomy, a thin, transparent membrane was observed. The cyst was punctured dorsally and found to contain a clear, cerebrospinal fluid-like liquid. After exposing the dural plane and the resection of a small portion of the cyst wall, the cyst was released toward the ventral side. Ultimately, communication was established between the cyst and the subarachnoid space on both the dorsal and ventral sides. Duroplasty was performed using a dural substitute and 6-0 vascular Prolene, reinforced with fibrin glue. Hermetic closure was confirmed by performing a Valsalva maneuver. The diagnosis of an arachnoid cyst was confirmed based on intraoperative findings, although a pathological diagnosis could not be established due to the limited amount of tissue available. No intraoperative complications occurred, and neurophysiological monitoring remained stable throughout the procedure.

Postoperatively, the patient showed improvement in right lower limb strength. He remained on bed rest for the first 24 h, with the surgical wound remaining clean and dry. Active mobilization was initiated on postoperative day 2.

Initial attempts at urinary catheter removal were unsuccessful. However, with intermittent catheterization, spontaneous voiding progressively resumed, and the patient achieved complete urinary independence by postoperative day 4.

**Figure 3:**
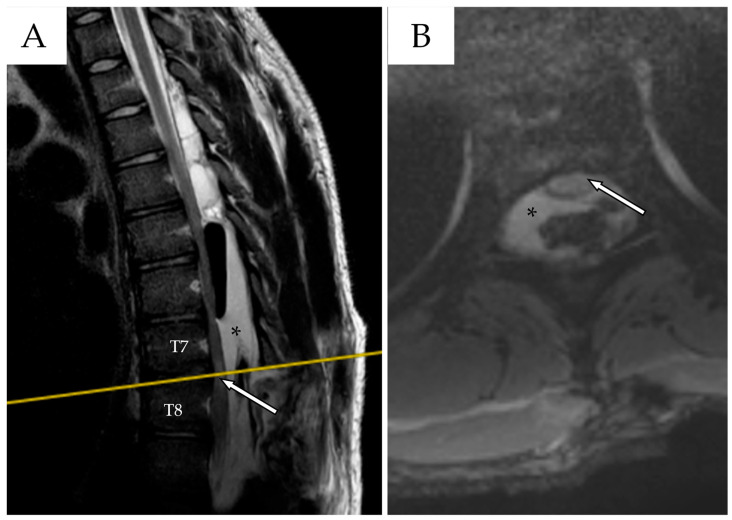
(**A**) Postoperative MRI, sagittal section, TSE T2-WI. After T8 laminectomy with cyst fenestration, the spinal cord appeared less compressed at the T7–T8 level (white arrows with black outlines). Fenestration was confirmed by the presence of an air sac within the caudal portion of the SAC (black asterisk). The yellow line indicates the level at which the axial section in (**B**) was obtained. (**B**) The spinal cord, although not yet re-expanded, appeared less compressed.

A postoperative MRI demonstrated a mild reduction in cyst volume and clear spinal cord decompression (Figure 3). The patient was enrolled in outpatient rehabilitation and was discharged on postoperative day 10. Upon discharge, the patient demonstrated independent ambulation with minimal aid. Right knee paresthesia had completely resolved. A motor deficit of the right foot persisted, predominantly affecting dorsiflexion (MRC 3/5), with mild residual weakness in plantarflexion.

### 2.2. First Follow-Up

**Figure 4:**
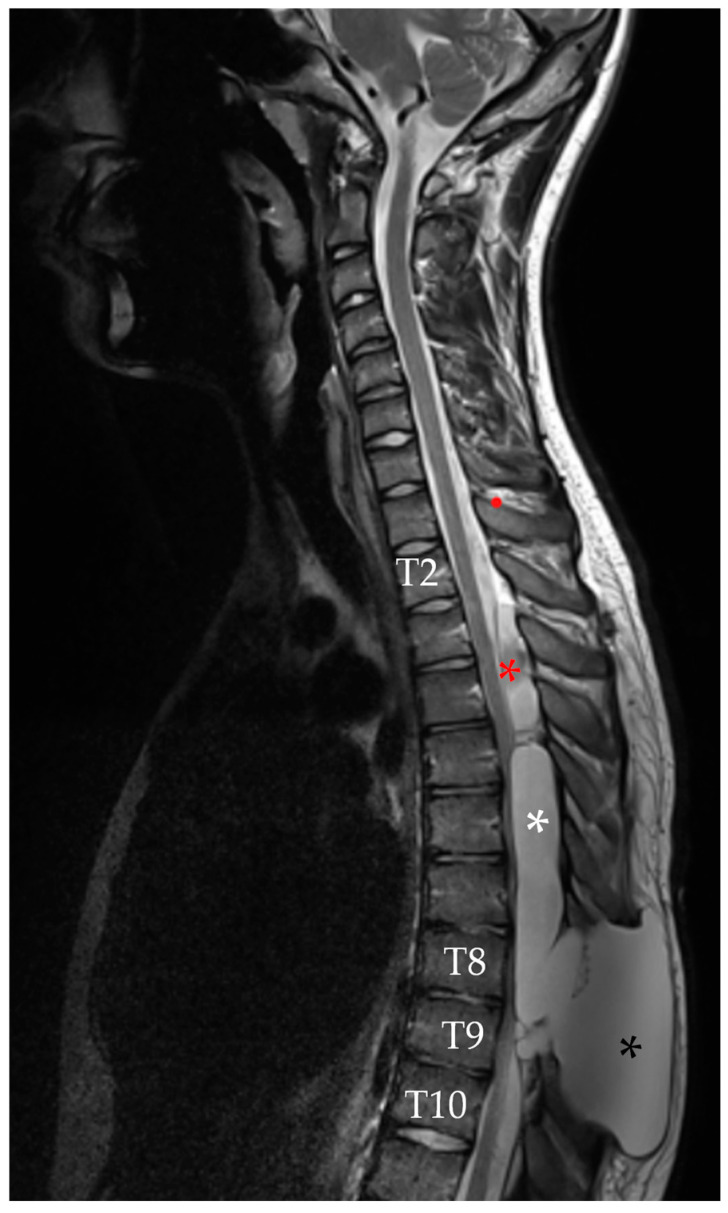
First follow-up MRI, sagittal section, TSE T2-WI. Both the cranial (red asterisk) and caudal (white asterisk) components of the SAC appear enlarged compared to the MRI in Figure 3, especially the latter. Additionally, a new pseudomeningocele (black asterisk) is visible at the level of the previous fenestration.

At the routine outpatient follow-up visit two weeks after discharge, urinary function had fully recovered. However, the control thoracic spine MRI revealed enlargement of the residual cystic components, both cranially at T2–T4 and caudally at T8–T9, adjacent to the previous fenestration site. Additionally, an 8.5 cm pseudomeningocele was identified, along with spinal cord signal changes between T5 and T8 (Figure 4). Given the clinical stability despite the MRI findings, a decision was made to continue close follow-up, with repeat imaging scheduled in two weeks.

At four weeks post-discharge right leg strength had improved to MRC 4/5, but a mild weakness developed on the left side (MRC 4/5). The patient also experienced new onset bladder tenesmus. The follow-up MRI with CSF flowmetry demonstrated no communication between the intradural space and the extradural cystic lesion. Given the MRI findings, outpatient myelography was scheduled, followed by clinical and radiological reassessment.

### 2.3. Second Admission

**Figure 5:**
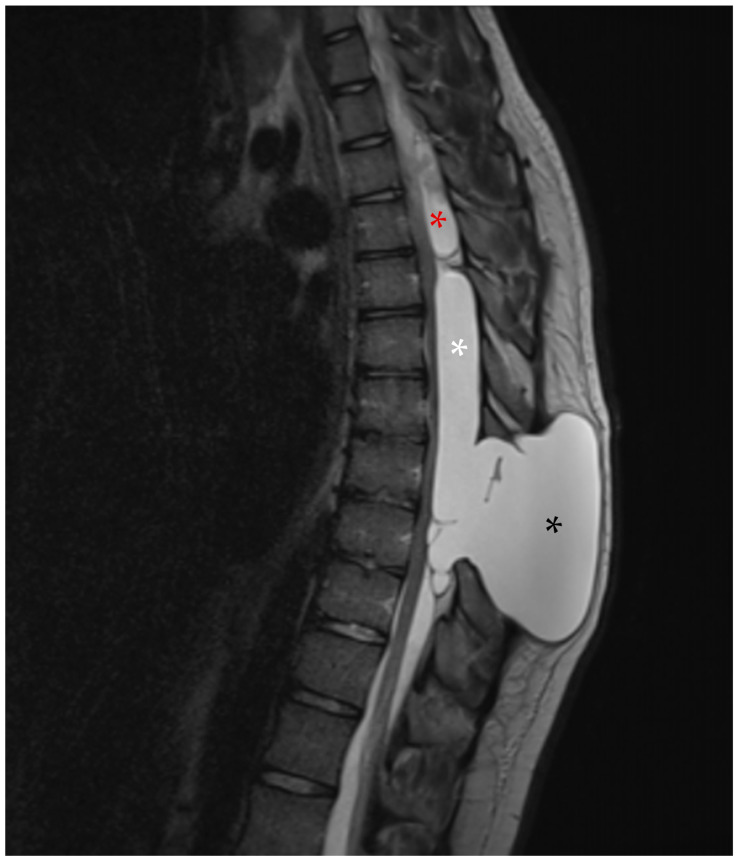
Emergent MRI, sagittal section, TSE T2-WI. Although the cranial component (red asterisk) remained stable, the caudal component (white asterisk), along with the pseudomeningocele (black asterisk), had enlarged and appeared under tension, which could explain the onset of the patient’s symptoms.

However, in early April, the patient returned to our emergency department with worsening of the bilateral lower limb weakness, gait disturbance, and urge incontinence. Neurological examination revealed bilateral leg weakness (M2 on the right, M3 on the left, globally), bilateral Babinski signs, and preserved perineal sensation. An emergent MRI was consistent with a further enlargement of the cyst and of the pseudomeningocele (Figure 5).

A second surgery was performed, again with continuous intraoperative neurophysiological monitoring. The procedure included repeat cyst fenestration, pseudomeningocele drainage, and placement of an external spinal CSF shunt. The latter was positioned to help control the volume of the pseudomeningocele and to promote wound healing, as the surgical site had already been reopened twice. CSF cultures obtained intraoperatively were negative for infection.

**Figure 6:**
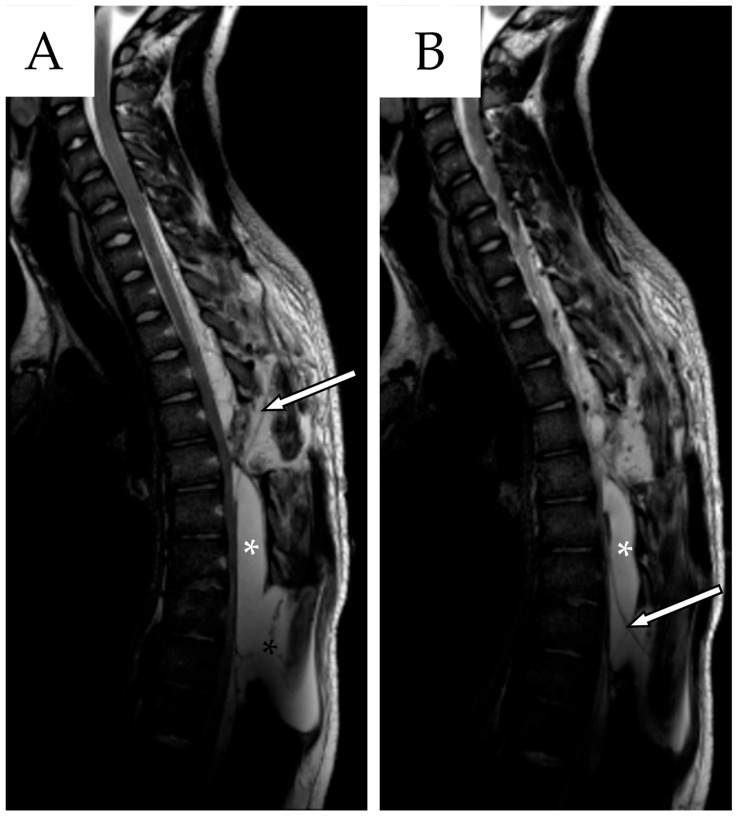
(**A**,**B**) Immediate post-second surgery MRI. The external drainage (white arrows with black outlines) is visible along the length of the SAC (white asterisk); it was inserted at the T4–T5 level and exits at the level of the pseudomeningocele (black asterisk), designed to drain as much CSF collection as possible.

The immediate postoperative MRI showed minimal reduction in cyst volume (Figure 6). The shunt had a steady output of about 10 mL/h, and was removed on postoperative day 5. A new MRI performed at two weeks after surgery showed cranial enlargement of the fluid collection with persistent spinal cord compression. Myelography identified two CSF fistulas: one at the T3–T4 level communicating with the subarachnoid space, and another at T8–T9 connecting to the pseudomeningocele.

Following multidisciplinary consultation with the interventional neuroradiology team, a targeted epidural blood patch under CT guidance was performed, leading to the apparent exclusion of the cyst and radiographic closure of both fistulous tracts.

**Figure 7:**
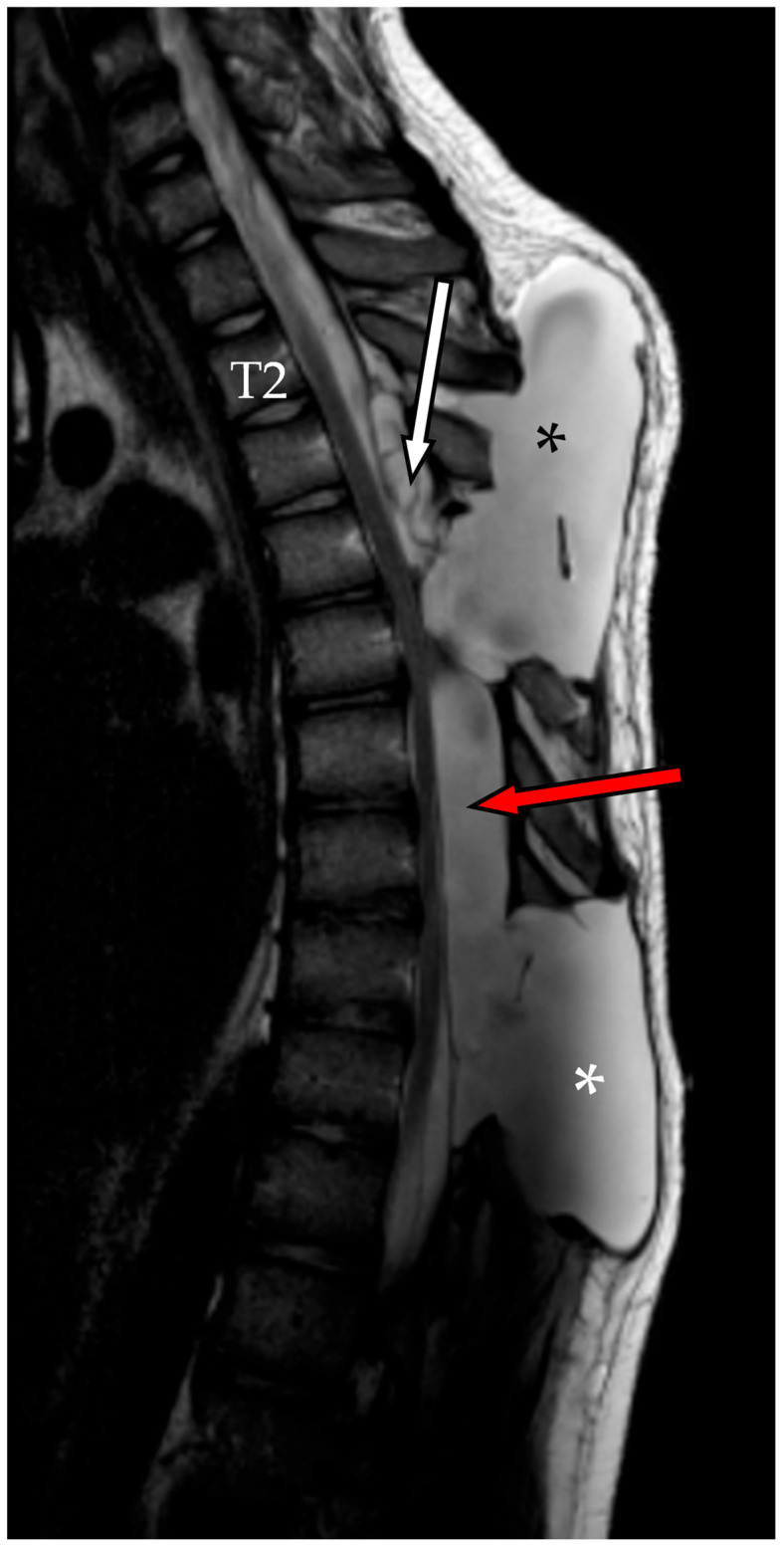
Control MRI after percutaneous blood patch procedure. The cranial component of the SAC remained stable (white arrow with black outlines), while the caudal component (red arrow with black outlines), along with the pseudomeningocele (white asterisk), was refilled and slightly enlarged. At the T4–T5 level, a new pseudomeningocele (black asterisk) has appeared, extending into the soft tissues from T2 to T5.

Although the blood patch was successfully performed, follow-up imaging at two weeks revealed the onset of a new cystic formation at the T4–T5 level extending in the soft tissues from T2 to T5 compatible with another pseudomeningocele; it probably developed at the point of entrance of the previously placed external drainage. The cranial component of the SAC was stable, whereas the caudal component together with the caudal pseudomeningocele appeared refilled and slightly enlarged (Figure 7). The patient’s neurological status remained stable though and a conservative management of “watch and see” was adopted.

During this period lasting three weeks, the pseudomeningocele collection continued to increase in size, and was appreciable at the inspection evaluation. MRI confirmed ongoing spinal cord compression at the T7–T8 level, and myelography demonstrated persistent cerebrospinal fluid leakage from a dorsal fistula at T3–T4.

**Figure 8:**
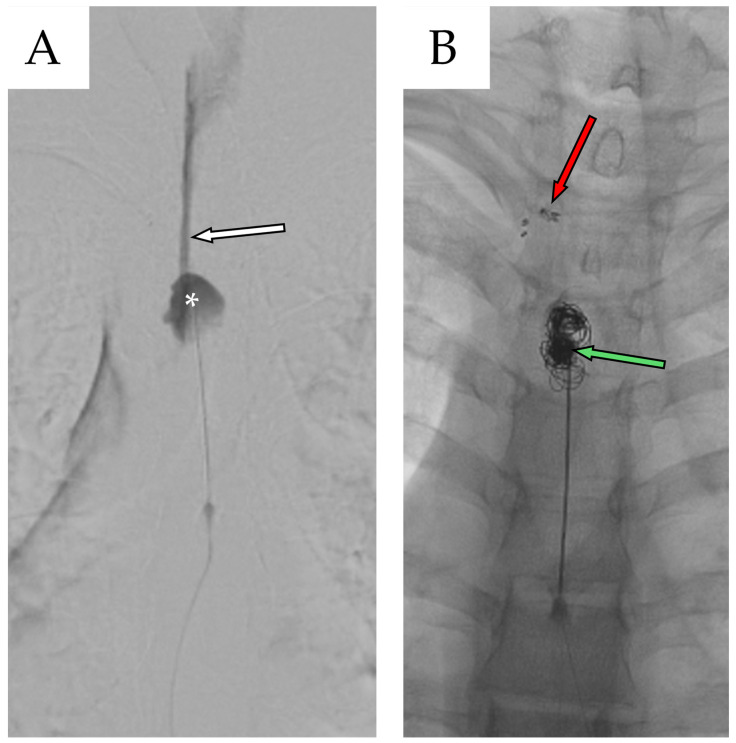
(**A**) Preoperative fluoroscopic myelography after contrast agent infusion shows the caudal portion of the cyst (white asterisk), along with the fistulous stream originating from the overlying fistulous tract (white arrow with black outline). (**B**) Postoperative fluoroscopic myelography confirms closure of the fistula at the T3–T4 level using metal clips (red arrow with black outline), while the remaining subarachnoid cavity (SAC) is filled with metallic coils and blood (green arrow with black outline).

A new multidisciplinary consultation was convened to address the need for definitive surgical repair. Two months after second admission, the patient underwent a third and final surgical procedure, consisting of bilateral T4 laminectomy with clipping of the T3 fistulous tract combined with percutaneous repair of the dural leak using metallic coils placed within the cystic cavity followed by a new blood patch (Figure 8).

**Figure 9:**
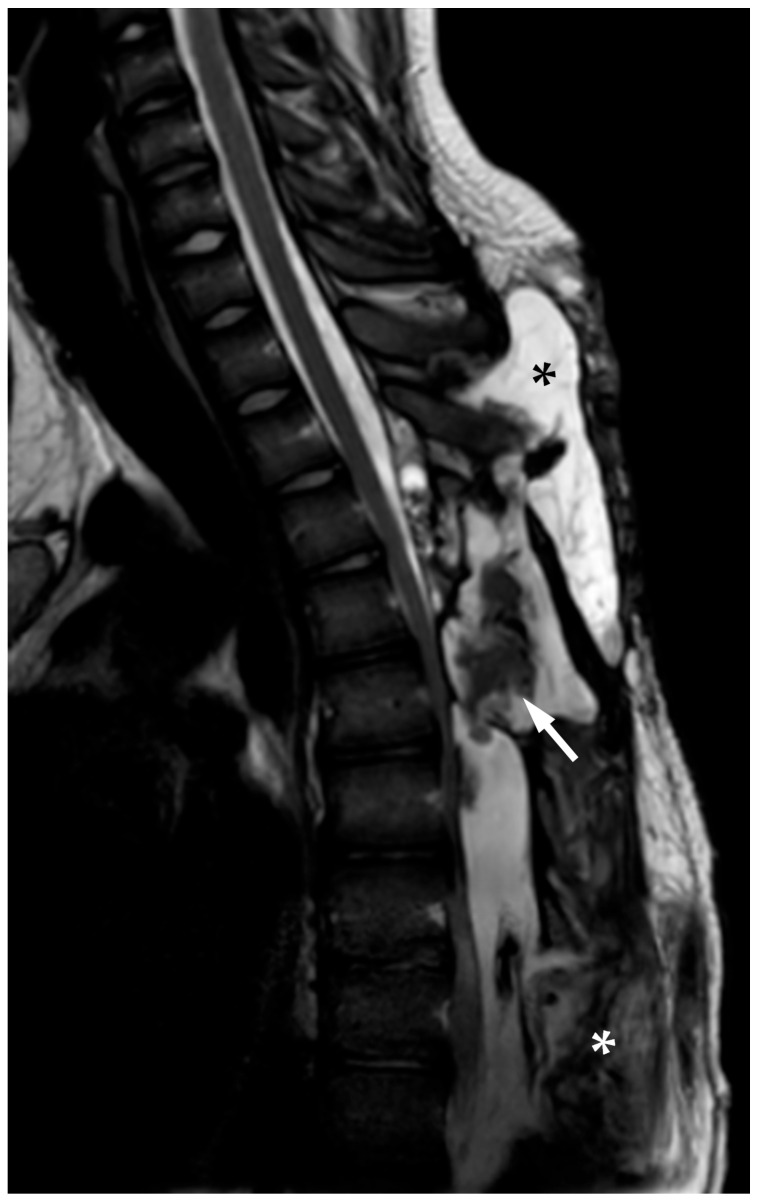
Sagittal T2-weighted MRI performed 7 days after the last surgery, which included percutaneous repair of the dural leak. The result of the blood patch is visible in the upper portion of the spinal arachnoid cavity (white arrow). A marked reduction in the cranial pseudomeningocele (black asterisk) is noted, along with near-complete resolution of the inferior pseudomeningocele (white asterisk).

Finally, MRI at 1 week after surgery showed a reduction in size of the pseudomeningocele at the T2–T4 level, along with decreased overall spinal cord compression and almost complete resolution of the caudal pseudomeningocele (Figure 9). The patient remained neurologically stable with mild improvement and no new deficits were observed.

The patient was subsequently discharged to their home. At discharge, the patient presented with residual motor deficits in the lower extremities, with muscle strength graded as MRC 4+/5 on the left and MRC 4−/5 proximally and 4/5 distally on the right. No sensory deficits were noted across thermal-nociceptive, light touch, or discriminatory modalities (both epicritic and protopathic). Bladder function was preserved, and bowel transit remained normal. Patellar reflexes were hyperelicitable, with an expanded reflexogenic zone. Independent ambulation was achieved, with partial weight-bearing allowed on the more affected limb, although gait remained slightly circumductory.

### 2.4. Second Follow-Up

The patient remained in good clinical condition, with no further neurological deterioration. He underwent serial follow-up evaluations every three months during the first year, including both clinical assessments and control MRI scans. These demonstrated a gradual regression of radiological signs of myelopathy and a reduction in the size of the pseudomeningocele. He continued occupational and physical therapy and was educated on strict return precautions and warning signs (i.e., “red flag” symptoms). The patient is currently undergoing regular outpatient follow-up for ongoing monitoring, now scheduled annually.

## 3. Discussion

According to the classification proposed by Nabors et al. [1], spinal arachnoid cysts (SACs) are categorized into three types: Type I, II, and III. Type I cysts are extradural without involvement of nerve root fibers; Type II cysts are extradural with involvement of nerve root fibers; and Type III cysts are intradural. In the present case report, we describe a Type I cyst. The origin of the lesion remains unclear: its size and radiological appearance suggest a congenital SAC, whereas the onset of symptoms following physical exertion could indicate a secondary SAC. Nevertheless, the congenital origin appears more likely.

Congenital SACs are thought to originate either from diverticula of the septum posticum (anatomic theory) [2] or from hypertrophic and dilated arachnoid granulations [3,4]. These congenital cysts are sometimes associated with central nervous system malformations, such as syringomyelia [5], not presented in our case. Secondary SACs may result from subarachnoid hemorrhage [6], trauma, infection, neoplasms, arachnoiditis, or iatrogenic causes, including diagnostic procedures such as myelography and, in rare cases, post-laminectomy changes [7,8].

Type I spinal arachnoid cysts, which are extradural and do not involve nerve root fibers, are more common than intradural cysts (Type III) and Type II extradural cysts. They are most often located in the thoracic spine, typically posterior or posterolateral to the spinal cord, as seen in the present case, but they may also occur in the cervical and lumbar regions [3,9]. While primary extradural SACs (including Type I) are thought to be congenital and dorsally located, secondary cysts may present ventrally and can involve a valve-like mechanism that disrupts CSF dynamics [10].

The pathogenesis of spinal arachnoid cysts (SACs) remains incompletely understood, in part due to the still unclear mechanisms regulating cerebrospinal fluid (CSF) dynamics and pressure within the central nervous system [11]. Several theories have been proposed to explain both the development and progressive enlargement of these cysts. According to the ‘ball-valve’ mechanism described by Rohrer [12], once an arachnoid protrusion forms, CSF becomes trapped within the cyst without adequate drainage. The ‘osmotic enlargement’ theory suggests that hyperosmolar cyst fluid may draw water into the cyst via osmotic gradients [13]. A third hypothesis posits that the cyst walls themselves may secrete fluid [14].

Extradural SACs may originate from arachnoid herniation through dural defects, whereas intradural cysts are thought to result from alterations of the arachnoid trabeculae [15]. In general, any mechanism that increases CSF pressure may contribute to cyst growth.

There appears to be no clear gender or age predilection reported in the literature [4,5]. However, when symptomatic, SACs most commonly present in early adulthood, typically between the ages of 30 and 50 [2]. Earlier onset has been reported in some cases—for example, in the case series by Abdel-Hameed & Morsy [7], which included a patient as young as 3 years old—and our case again shows its unicity for the young age of onset.

Magnetic resonance imaging with contrast is the modality of choice for diagnosing spinal arachnoid cysts. On T2-weighted sequences, SACs appear as lesions isointense with CSF and are best visualized on sagittal views [7]. MRI allows for precise localization of the lesion and can reveal internal features such as septations or loculations [4].

MRI also plays a critical role in postoperative evaluation, particularly in assessing residual spinal cord compression. Its high spatial resolution enables detailed characterization of the cyst and aids in the differential diagnosis from other lesions, such as neurenteric cysts (commonly located in the cervicothoracic region), perineural cysts, synovial cysts, epidermoid cysts, and dermoid cysts [7].

Currently, there are no definitive guidelines for the treatment of SACs. In general, surgical intervention is indicated in symptomatic cases, particularly in the presence of myelopathy; all criteria for operation were presented by our patient [3].

For patients with mild or minimal symptoms, a conservative approach with clinical monitoring may be appropriate, with surgery reserved for those who experience significant neurological deterioration [16]. According to the literature, delaying surgical treatment in mildly symptomatic patients—even in the event of later clinical progression—does not appear to adversely affect long-term neurological outcomes [17].

Treatment options include surgical excision, fenestration, and cysto-subarachnoid shunting. The choice of procedure depends on the cyst’s location, type, size, and the degree of adhesion to surrounding structures [18]. Complete surgical excision is considered the gold standard, as it is associated with lower recurrence rates and improved long-term clinical outcomes, while most reported recurrences have occurred in patients who underwent fenestration alone [9,18]. As previously said, the choice of undergoing a fenestration in the myelopathic level instead of an excision was based on the young age of the patient and the cranial to caudal extension of the SAC itself, which would have needed a long incision and a wide decompression with the need of thoracic spine stabilization with fixation from T1 to T9.

Identifying the precise communication point between the cyst and the subarachnoid space is essential to minimize surgical exposure, reduce the risk of postoperative CSF leakage, and prevent pseudomeningocele formation [19].

Intraoperatively, distinguishing the cyst wall from the surrounding arachnoid membrane can be challenging. This often precludes en bloc resection and increases the risk of leaving residual tissue, thereby contributing to recurrence. The use of intraoperative dyes has been proposed to facilitate complete and safe excision by delineating the cyst boundaries from adjacent arachnoid tissue [20]. Additionally, staining the SAC may assist in defining its margins, potentially avoiding unnecessary laminectomies or dural incisions that could increase the risk of postoperative complications such as infection, hemorrhage, or spinal instability.

To minimize surgical trauma, endoscopic techniques may be utilized to localize the site of CSF leakage, thereby enabling more targeted laminectomies and reducing the risk of postoperative complications [3].

In all surgical procedures performed on our patient—including in the emergency setting—intraoperative neurophysiological monitoring (IONM) was employed to minimize the risk of spinal cord injury. This was made possible by the availability of dedicated neurophysiologists and IONM technicians at our institution.

As expected—and consistent with the higher recurrence rate associated with fenestration alone—the patient experienced a recurrence of symptoms after initial postoperative improvement, necessitating revision surgery. The clinical course was further complicated by the development of a significant pseudomeningocele, a known complication following arachnoid cyst surgery, particularly when the fistulous tract cannot be clearly identified. Fortunately, to date, no CSF infection has been observed in the present case.

In cases of recurrence, cystoperitoneal, cystopleural, or cystoatrial shunting may be considered as alternative treatment options [21,22]. In our case, a cystoperitoneal shunt was proposed during the second multidisciplinary discussion as a definitive solution. However, the decision was ultimately made to forgo this approach, due to concerns about placing a permanent silicone device in a 17-year-old patient and the associated risk of shunt-related infections—particularly in the context of an existing pseudomeningocele, which may further increase infection risk.

In the present case, a novel technique was implemented, combining closure of the fistulous tract using titanium aneurysmal clips with the placement of metallic coils inside the SAC. To the best of our knowledge, no similar attempts have been previously reported in the available literature. The rationale behind the combination of a blood patch and coiling was that a blood patch alone would probably not have been sufficient to seal the SAC, as the presence of the pseudomeningocele and the high volume of CSF would have diluted the blood, preventing the coagulation mechanism from taking place. By inserting the coil together with the blood patch, a pro-thrombotic surface is provided to help trigger the coagulation cascade.

Neo M. et al. [23] described in 2004 the closure of a fistulous tract in an extradural spinal arachnoid cyst using metal clips, resulting in an almost complete disappearance of the cyst just 12 days after the operation. The underlying principle is the same: to close the communication between the subarachnoid space and the cyst. However, in our case, platinum coiling was added to promote coagulation of the cyst’s contents, in combination with a new percutaneous blood patch.

The combined approach proved effective, though the results were not as striking as those reported by Neo M. et al., likely due to the presence of the pseudomeningocele. Nevertheless, the patient achieved a satisfactory neurological outcome and favorable imaging findings at the last outpatient follow-up, one year after discharge.

To further characterize the SACs and contextualize the present case within the existing literature, we conducted a comprehensive review of case reports and case series published between 2005 and 2024. A total of 37 articles describing the clinical history of 157 patients with spinal arachnoid cysts were identified.

Based on the collected data, this pathology predominantly affects a younger population, with most patients ranging between 20 and 50 years of age. However, pediatric cases (as young as 4 years) and elderly patients (up to 80 years) were also reported. The mean age across cohorts ranged between 8 and 60 years in larger studies. A slight female predominance was observed (approximately 85 females vs. 72 males).

Risk factors were identified in a subset of patients, mainly including spinal trauma, prior spinal surgery, central nervous system anomalies, and, less frequently, epidural steroid injections, schwannoma, or post-surgical meningitis.

The thoracic spine was the most commonly affected region, followed by the Thoracic–lumbar and lumbar regions. Less frequently, cysts were located in the cervical or Cervical–thoracic spine. Multisegmental involvement was observed in rare cases.

Regarding cyst classification, Type III (intradural or intramedullary cysts) was the most frequent, accounting for the majority of cases. Some articles reported mixed-type cysts or unspecified classification.

Symptoms varied depending on cyst location and size. The most common clinical presentations were motor weakness, particularly in the lower limbs, urinary and/or bowel dysfunction, and radicular pain. Sensory deficits, gait ataxia, myelopathy, and in some pediatric cases, tetraparesis or quadriplegia, were also noted. A few cases were asymptomatic and discovered incidentally.

Surgical treatment was the mainstay in nearly all symptomatic cases. Complete excision was considered the gold standard and was performed in most patients, often with good outcomes and remission of symptoms. In other cases, fenestration alone or followed by a new intervention of excision was used. Less frequently, shunt placement or conservative management was adopted in selected asymptomatic or high-risk patients.

Postoperative outcomes were generally favorable. The majority of patients showed full or significant neurological recovery, even in those requiring reoperation for complications such as recurrence, CSF fistulas, or pseudomeningoceles. Only a small subset experienced persistent or progressive symptoms. All data are summarized in Table 1 and Table 2.

## 4. Conclusions

This case highlights both the diagnostic challenges and therapeutic complexities associated with spinal arachnoid cysts, particularly in young patients. Despite initial surgical management through fenestration, recurrence and complications such as pseudomeningocele necessitated innovative treatment strategies. The implementation of a novel technique combining aneurysmal clipping and platinum coiling, along with a percutaneous blood patch, represents a promising approach in selected cases where standard surgical options are limited or pose significant risks. While the outcome was ultimately satisfactory, the experience underscores the importance of individualized treatment planning, multidisciplinary collaboration, and the need for continued research into less invasive and more effective management options for SACs. Further studies and long-term follow-up will be essential to validate the efficacy and safety of such combined interventions.

## Figures and Tables

**Table 1:** Summary of the literature review data (first part). * Age refers to the age at the time of surgery (in years); for case series, the median age is reported. ** When referring to Type I cysts, we considered only Type Ia, as Type Ib cysts are outside the scope of this study. NA: Not Available. LBP: Low Back Pain. TLIF: Transforaminal Lumbar Interbody Fusion.

Author	Country/Year	N°	Age *	Gender	Risk Factors	Location of the Cyst	Type of Cyst **	Symptoms	Management
Ichinose T et al. [24]	Japan, 2020	1	4	M	No	Cervical	Type III	Tetraparesis, bladder/bowel dysfunction	Fenestration
Novak L et al. [25]	Hungary, 2005	1	15	F	Spine trauma	Thoracic	Type I	Spastic paraparesis inferior limbs, sensory loss	Excision
Lee HG et al. [26]	Korea, 2019	1	50	M	Spine surgery	Lumbar	Type III	LBP, right S1 radiculopathy	Fenestration, excision
Krstačić A et al. [27]	Croatia, 2016	1	50	F	No	Lumbar	Type III	LBP, right S1 radiculopathy, right leg hyposthenia	NA
Lee & Cho [28]	China, 2001	3	8, 9, 9	M, M, F	No, No, No	Thoracic–lumbar, cervical–thoracic, thoracic	Type III	1: Cauda equina 1: Spastic gait 1: Quadriplegia	Fenestration, excision
Bond AE et al. [29]	USA, 2012	31	6, 9 (median)	17 F, 14 M	16 CNS anomalies	11 thoracic, 2 lumbar, 3 sacral, 4 cervical–thoracic, 4 thoracic–lumbar, 4 lumbar–sacral, 2 cervical	19: Type III 11: Type I, 1: Type III/I	21: Radiculopathy/myelopathy 3: Asymptomatic	29: Excision 2: Fenestration, excision
Liu JK et al. [30]	USA, 2005	1	11	F	No	Thoracic	Type I	Bladder dysfunction, gait ataxia, lower limbs hyposthenia	Excision
El-Hajj VG et al. [31]	Sweden, 2023	1	35	F	Surgery for cranial arachnoid cyst	Cervical–thoracic	Type III	Neck and back pain, upper limbs weakness	Excision
Özdemir M et al. [32]	Turkey, 2019	1	22	M	No	Cervical–thoracic	Type I	Neck pain, numbness and weakness of the left upper arm	Excision
Ebot J et al. [33]	USA, 2020	1	80	F	No	Thoracic	Type III	Lower extremity weakness, decreased sensation and hyperreflexia	Fenestration
Alugolu R et al. [34]	India, 2016	1	54	F	No	Thoracic	Type III	Spastic weakness of lower limbs, decreased sensations below T12	Excision
Nayak R et al. [35]	India, 2015	1	7	F	No	Thoracic	Type I	Numbness and weakness of lower limbs	Excision
Sharif S et al. [36]	Pakistan, 2017	1	25	M	No	Lumbar	Type III	LBP, numbness of lower limbs, cauda equina	Excision
Lin & Jason [37]	Malaysia, 2018	1	35	M	No	Thoracic	Type I	Numbness and weakness of lower limbs	Excision
Shaaban A et al. [38]	Quatar, 2019	1	31	M	No	Thoracic	Type III	T6 sensory level, LBP, bladder/bowel disfunction	Excision
Raes & Oostra [39]	Belgium, 2021	2	38, 45	F, M	Spine trauma with T12 and L1 fracture	Thoracic	Type III	Loss of strength of lower limbs, sensory disturbances in the right leg, paraplegia and T11 neurological level	Fenestration
Taccone MS et al. [40]	Canada, 2018	1	72	M	TLIF L4-L5	Lumbar	Type III	Lower limbs weakness in first day after surgery	Fenestration
Turel MK et al. [41]	USA, 2017	1	36	F	Epidural steroid injections	Lumbar	Type III	Asymptomatic	Conservative managed
Kong WK et al. [42]	Korea, 2013	1	65	M	Spine trauma	Thoracic–lumbar	Type I	Weakness of lower limbs, bladder dysfunction	Fenestration
Fischer KM et al. [43]	USA, 2022	1	7	F	No	Thoracic	Type III	Urinary and fecal incontinence	Excision
Sharma R et al. [44]	India, 2023	1	16	M	No	Cervical–thoracic–lumbar	Type I	Weakness of lower limbs, numbness of the right leg	Excision
Engelhardt J et al. [45]	France, 2015	1	18	M	No	Cervical	Type III	Cervical pain, paresthesia and weakness of both arms	Excision
Zekaj E et al. [46]	Italy, 2016	1	47	F	No	Thoracic	Type III	Right dorsal radicular pain, weakness of the left lower limb	Fenestration
Watanabe A et al. [47]	Japan, 2019	1	37	F	No	Thoracic	Type III	Abdominal pain, numbness and weakness of both lower extremities	Excision
Cavalcante-Neto JF et al. [48]	Brazil, 2021	1	38	F	No	Thoracic–lumbar	Type I	Transient weakness of lower limbs, bilateral patellar hyperreflexia	Excision
Marrone S et al. [49]	Italy, 2022	1	70	M	No	Thoracic	Type I	Bilateral lower limb weakness and paresthesia	Fenestration, excision
Alanazi RF et al. [50]	Saudi Arabia, 2022	1	47	F	No	Thoracic–lumbar	Type I	Progressive lower extremity paraparesis	Excision
Sawaya J et al. [51]	USA, 2024	1	15	F	No	Thoracic–lumbar	Type I	LBP radiating bilateral in posterior thighs and knees	Fenestration, excision
Ayantayo TO et al. [52]	Nigeria, 2023	1	49	M	No	Thoracic	Type III	Gait instability, urinary retention and paraplegia	Excision
Werner C et al. [53]	USA, 2020	1	58	M	Spinal schwannoma Post-surgical meningitis	Thoracic	Type III	Paraplegia and loss of sensation below T10, urinary retention	Fenestration
Pillai MK [54]	Oman, 2016	1	29	F	No	Cervical–thoracic	Type III	Posterior column dysfunction	Fenestration
French H et al. [55]	Australia, 2017	10	60 (mean)	F:M 2:1	NA	Thoracic	Type III	Thoracic myelopathy, gait ataxia 3: thoracic radicular pain 1: sphincter dysfunction	6: Fenestration 4: Excision
Shi L et al. [56]	China, 2021	41	41 (mean)	24F, 17M	NA	Thoracic–lumbar	Type I	Radicular pain, gait ataxia, myelopathy 1: cauda equina syndrome	Excision
Yılmaz E et al. [57]	Turkey, 2023	8	8 (mean)	4F, 4M	NA	Thoracic–lumbar	Type III	Motor weakness, urinary incontinence, sensory disturbance	7: Excision 1: Shunt placement
Tokmak M et al. [58]	Turkey, 2024	10	43 (mean)	6F, 4M	NA	Thoracic–lumbar	Type I	Numbness and progressive weakness of the lower extremities, back pain	9: Excision 1: Conservative managed
Menezes AH et al. [59]	USA, 2017	2	14, 34	M, F	Back trauma	Thoracic–lumbar, lumbar	Type I	Gait instability, urinary retention, pain in the left leg	Excision
Kalsi P et al. [60]	Canada, 2022	11	47 (mean)	3F, 8M	3 presented with associated syrinx	10 cervical–thoracic, 1 cervical	Type III	Neuropathic pain, back pain, weakness, gait and balance problems, sensory issues, sphincter disturbance, radicular pain, long tract signs, gait ataxia	10: Fenestration 1: Excision 1: Shunt placement

**Table 2:** Summary of the literature review data (second part).

Improvement	Relapse and Complications
No	No
Yes	No
Yes	NA
NA	No, no, no
2: Yes 1: Partial	1: Fistula 1: Pseudomeningocele 1: Relapse and shunt
27: Yes 4: No	No
Yes	Yes
Yes	No
Yes	No
Yes	Pseudomeningocele
Yes	No
Yes	No
Yes	No
Yes	No
Yes	Yes, yes
Yes, yes	No
Yes	NA
NA	No
Yes	No
Yes	No
Yes	No
Yes	Yes
Yes	No
Yes	No
Yes	No
Yes	No
Yes	No
Yes	No
Yes	Yes
Yes	No
Yes	1: Pseudomeningocele 1: Syrinx
Yes	2: Yes
Yes	No
Yes	1: Fistula
Yes	No
Yes	No
4: No 7: Yes	Yes

## Data Availability

Due to patient privacy concerns, the data presented in this study are available from the corresponding author upon reasonable request.

## Abbreviations

The following abbreviations are used in this manuscript:

SACs	Spinal Arachnoid Cysts
CSF	Cerebrospinal Fluid
MRI	Magnetic Resonance Imaging
MRC	Medical Research Council
ceMRI	contrast-enhanced Magnetic Resonance Imaging
IONM	Intraoperative Neurophysiological Monitoring
TSE	Turbo Spin Echo
WI	Weighted Imaging
NA	Not Available
LBP	Low Back Pain
TLIF	Transforaminal Lumbar Interbody Fusion
